# Retraction: Yang, B. et al. Nitrogen Doped Intercalation TiO_2_/TiN/Ti_3_C_2_T_x_ Nanocomposite Electrodes with Enhanced Pseudocapacitance. *Nanomaterials* 2020, *10*, 345

**DOI:** 10.3390/nano11040936

**Published:** 2021-04-06

**Authors:** Ben Yang, Yin She, Changgeng Zhang, Shuai Kang, Jin Zhou, Wei Hu, Nanomaterials Editorial Office

**Affiliations:** 1 Key Laboratory of Optoelectronic Technology and System of Ministry of Education, College of Optoelectronic Engineering, Chongqing University, Chongqing 400044, China; 201908021058@cqu.edu.cn (B.Y.); SillerZ@cqu.edu.cn (C.Z.); 2 Key Laboratory of Fundamental Science Micro/Nano Device System Technology, Micro System Research Center of Chongqing University, Chongqing 400044, China; 3 Intelligent Manufacturing Technology Institute, Chongqing Institute of Green and Intelligent Technology, Chinese Academy of Sciences, Chongqing 400714, China; Kangshuai@cigit.ac.cn; 4 Chongqing Academy of Metrology and Quality Inspection, Chongqing 401121, China; zhoujin1203@163.com; 5 St. Alban-Anlage 66, 4052 Basel, Switzerland

This article [1] has been retracted at the request of the authors due to data errors. In the investigation of the cycle performance of the electrode, the last five galvanostatic charge-discharge (GCD) curves in the inset of Figure 10a and the corresponding Nyquist impedance spectra in Figure 10b were not updated. As a consequence, the cycle performance was not accurately represented by the reported results. After much deliberation, the authors decide to withdraw this article.

The decision to retract has been taken in agreement with the authors and approved by the Editor-in-Chief.

The Nanomaterials Editorial Office and authors apologize to the readers of *Nanomaterials* for any inconvenience caused. To ensure the addition of only high-quality scientific work to the field of scholarly publication, this paper [1] is retracted and shall be marked accordingly. 
